# Supplementary figures and images for: Early postpartum HbA1c after hyperglycemia first detected in pregnancy—Imperfect but not without value
Source: PLoS One. 2023 Jun 8;18(6):e0282446. doi: 10.1371/journal.pone.0282446 (PMC10249808; doi:10.1371/journal.pone.0282446)

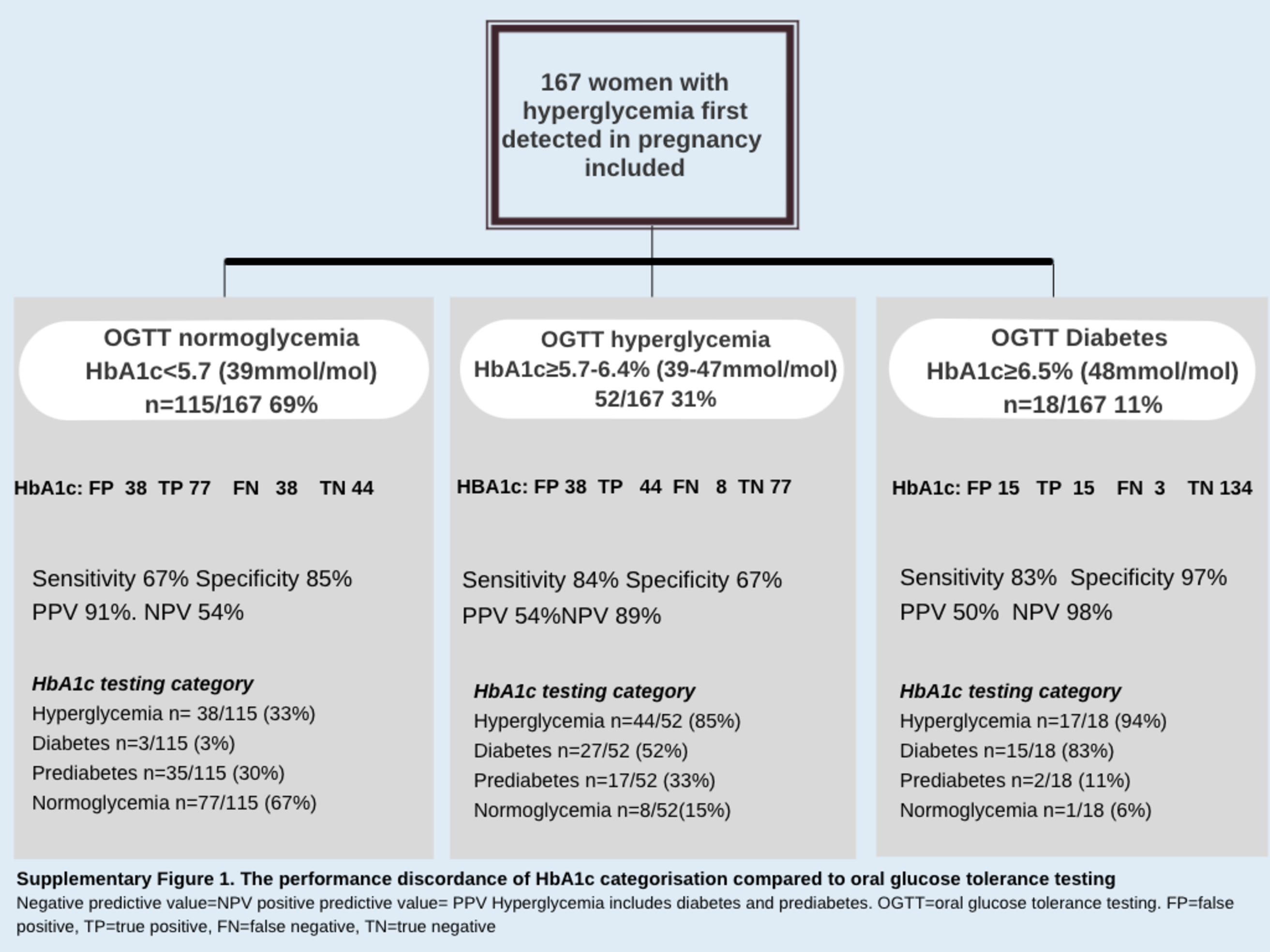

Supplement: S1 Fig — (TIF) [file pone.0282446.s001.tif]
