# Supplementary material for: Early postpartum HbA1c after hyperglycemia first detected in pregnancy—Imperfect but not without value
Source: PLoS One. 2023 Jun 8;18(6):e0282446. doi: 10.1371/journal.pone.0282446 (PMC10249808; doi:10.1371/journal.pone.0282446)
Supplement: Attachment — Submitted filename: Review PLoS ONE postpartum hbA1c.pdf [file pone.0282446.s002.pdf]

Thank you for the opportunity to review the manuscript “**Early postpartum HbA1c after hyperglycemia first detected in pregnancy – imperfect but not without value”**

This is an interesting novel study and should be published. However, some revisions are needed before this manuscript can be considered.

Keywords:

These are too long- consider following MeSH terms: hyperglycemia, pregnancy, gestational diabetes, HbA1c, postpartum, oral glucose tolerance test

Abstract:

- Results confusing – consider rewriting clarifying that hyperglycemia consists of prediabetes and diabetes. Maybe instead of using term “hyperglycemia”, divide into normoglycemia, prediabetes and diabetes?

Introduction:

- Generally well written.
- However, there is one MAJOR error that authors need to correct before this can be published: in the introduction, authors refer to “glycosylated” hemoglobin and in the discussion they mention “glycosylation”. GLYCOSYLATION is an enzymatic process, catalysed by glycosyltransferase, where a carbohydrate group is added to a protein. This is important for protein folding and activity, cell-to-cell adhesion, diversity of the proteome, etc and is a non-pathological process. However, GLYCATION refers to the non-enzymatic addition of a carbohydrate to a protein, is a pathological process and associated with glycaemia and aging. Many old papers refer to HbA1c as “Glycosylated haemoglobin” which is **incorrect**. Please correct introduction to “glycated haemoglobin” and discussion to “glycation”!
- HbA1c > 6.5% should be repeated for diagnosis of DM
- Non-glycemic factors affecting HbA1c should be mentioned e.g. iron status, illness, genetic and haematological factors and ethnicity

Methodology:

- Why was iron status / iron treatment not considered? Both common in pregnancy affect HbA1c.
- Four weeks postpartum may be too soon as may still reflect pregnancy HbA1c – should have been determined at 12 weeks minimum (red blood cell life span of 120 days)
- Authors should mention that lab accredited to ISO 15189 (2012) standards
- What instrument is glucose determined on?
- Should mention that internal and external quality control performed.
- Correct HbA1c method: Tina-quant ® HbA1c assay Generation 3 on the Roche Cobas 6000 ® (Roche Diagnostics, Mannheim, Germany). This assay is standardized according to IFCC and transferable to DCCT/NGSP.

Results

- Well-written but quite long
- P-values should be given for comparisons

Discussion:

- Generally well written
- Start discussion by listing key findings of the study without actually reseating the results
- Some areas need to be clarified e.g. page 19 mentions that “women with prediabetes and HbA1c
  >- 6.5% at higher risk for T2DM. Isn’t it DM with that HbA1c?

Tables:

- P-values should be given for comparisons

Minor:

- Check consistency – authors using American English, yet haemoglobin spelt “haemoglobin” in introduction.
- NHLS does not need to be written out in full again under Biochemistry
- ADA in methodology needs to be written out
- HbA1c subheading in methodology – small c
- Don’t write out T2DM in full again in discussion

- -
